# The Assessment of Resin-Based Composite Sealants’ Effectiveness in Arresting Non-Cavitated Dentin Carious Lesions (ICDAS 3)—A 12 Month Follow-Up Preliminary Study

**DOI:** 10.3390/medicina60050734

**Published:** 2024-04-28

**Authors:** Liana Beresescu, Alexandra Mihaela Stoica, Elena Stepco, Csinszka Andrea Kovacs-Ivacson, Alexandru Vlasa, Csilla Benedek, Gabriela Felicia Beresescu

**Affiliations:** 1 Faculty of Dental Medicine, University of Medicine and Pharmacy, Science, and Technology George Emil Palade, 540139 Târgu-Mureș, Romania; liana.beresescu@umfst.ro (L.B.); andrea.kovacsivacson@umfst.ro (C.A.K.-I.); alexandru.vlasa@umfst.ro (A.V.); csilla.benedek@umfst.ro (C.B.); gabriela.beresescu@umfst.ro (G.F.B.); 2 Faculty of Dental Medicine, The State University of Medicine and Pharmacy “Nicolae Testemitanu”, MD-2004 Chisinau, Moldova

**Keywords:** dentinal caries, carious lesions, resin-based sealant, dental sealants, pit and fissure sealant

## Abstract

*Background and Objectives*: The therapeutic management of carious lesions remains a significant focus for researchers, given their persistently high prevalence despite being largely preventable. This study aimed to compare the effectiveness of a composite resin-based sealant material in halting extended non-cavitated dentin carious lesions when used therapeutically versus preventively on caries-free teeth over a period of twelve months. *Materials and Methods*: out of the 236 children examined, 45 were excluded from the study due to non-compliance with the inclusion criteria. Thus, the study included 191 children aged 10–12 years, and 764 molars in total. *Results*: among these molars, 171 were caries-free (ICDAS II code 0), forming the Control group, while 180 molars were classified with an ICDAS II score of 3, forming the Study group. All molars were sealed and evaluated at 6- and 12-month follow-up intervals. Both intervals revealed statistically significant differences (*p* < 0.05) in sealant retention and carious lesion development between sound (ICDAS code 0) and decayed (ICDAS code 3) teeth. *Conclusions*: the findings did not support the effectiveness of sealants in halting non-cavitated dentin carious lesions classified as ICDAS II with code 3 compared to their preventive application in sound teeth classified as ICDAS II with code 0.

## 1. Introduction

Dental caries remains the most prevalent chronic disease in children, even though it is largely preventable. Untreated dental caries is still a global public health issue and poses a serious economic burden [1,2,3]. Due to this fact, a therapeutic approach to carious lesions has been and continues to be a major concern for researchers in the field. Until recently, carious lesions were managed following the conventional “drill and fill’’ concept, meaning the complete carious tissue removal and the replacement of the missing tooth structure with a filling [4]. This surgical approach to dental care which implicates the removal of all carious tissues as a standard part of the dental caries treatment, is no longer recommended [5,6]. The benefits of removing all carious dental structures have been called into doubt because of concerns about possible adverse effects such as weakening tooth structure and pulp damage, thus reducing the longevity of the restoration and of the restored tooth [6]. Additionally, this approach toward dental caries resulted from the historical belief that it is an “infectious” disease caused by specific bacteria inducing demineralization [7]. Therefore, caries lesion management must involve the excising of “infected” dental tissue [8]. It was assumed that surgically removing contaminated demineralized tissue would “cure” the tooth of dental caries. Furthermore, the conventional drill-and-fill concept was based on the erroneous philosophy that once a lesion was established, its further progress was inevitable unless lesion microorganisms were eradicated from the “infected” dental tissue [9].

In contrast to this, a more modern definition of dental caries is “a process which occurs in the biofilm, leading to an imbalance in the equilibrium between tooth mineral and biofilm fluids” [4]. According to this, the caries process is not simply a continual demineralization one, leading to cavitation, but rather a dynamic process characterized by alternating periods of demineralization and remineralization [10]. If the demineralization process dominates, the result is the loss of minerals, leading to the manifestation of caries lesions. When environmental conditions change and the re-deposition of minerals dominates, the result is that the lesion progression halts [11,12]. In recent decades, numerous studies, involving placing fissure sealants over caries lesions, selective and stepwise excavation and restoration, or even a lack of caries removal [13,14,15], conclude that caries lesions can be arrested provided that clinically biofilm-free conditions are maintained [16]. The actual objective in the management of caries lesions, focuses on controlling caries activity, while preserving hard dental tissue. Thus, nowadays, nonselective caries removal is considered overtreatment and is no longer recommended [6,17].

As a result, in recent years, the use of sealing materials has been extended for therapeutic purposes including managing pits and fissure caries lesions. This minimally invasive technique involves the application of a sealant material over the carious process, which will ultimately halt its progression [14,18]. In the case of carious lesions limited to enamel, numerous literature studies already demonstrate the possibility of arresting these lesions using this new technique and recommend the therapeutic application of sealants [19,20,21]. For non-cavitated carious lesions that have extended into dentin, opinions are divided regarding a therapeutic approach, raising numerous questions and controversies. Some researchers advocate for minimally invasive treatment in this case by applying the sealant material over the carious process, believing that it will be halted in its progression on the principle that as long as the sealant remains intact, the underlying carious process cannot progress. They believe that state-of-the-art adhesives can provide the adequate adhesion and retention of sealants even in dental structures that have undergone demineralization due to carious grafting [22]. However, the results of other studies refute the success of this method, suggesting the impossibility of stopping the progression of carious lesions because in this case, material retention is compromised, involving frequent resealing and the progression of the carious process [23]. The latest systematic review studies [24,25] conclude that the effectiveness of sealants in these cases is not well-documented, and the current information is qualitatively weak, requiring more concise studies to evaluate the progression/arrest of non-cavitated dentin carious lesions under sealant materials.

The purpose of this study was to evaluate and compare the effectiveness of a composite resin-based sealant material used for therapeutic purposes in arresting extended non-cavitated dentin carious lesions (ICDAS code 3) to its effectiveness when applied preventively on caries-free teeth (ICDAS code 0) over a period of twelve months.

## 2. Materials and Methods

This study was conducted from February 2023 to March 2024 at the Integrated Dental Medicine Center of the University of Medicine, Pharmacy, Sciences, and Technology in Târgu Mureș. Ethical clearance to conduct the study was obtained from the Ethical Research Committee of George Emil Palade University of Medicine, Pharmacy, Sciences, and Technology from Târgu Mureș no. 2064/09.02.2023. Patients were selected from several schools in the city of Târgu Mureș, with the assistance of the respective school administrations. The selection criteria were as follows: healthy and cooperative patients, aged between 10–12 years, who had not undergone general fluoride treatment. Non-cooperative patients, those with general medical conditions and/or specific medications, and those who had previously received or were currently receiving systemic fluoride treatment, were excluded from the study. Before the beginning of the examinations, parents or legal tutors of children invited to take part in the study were informed with respect to the approved protocol, and their written consent was obtained. All the examinations and procedures were performed by two experienced dentists. Prior to the clinical data collection, the dentists completed an ICDAS II calibration course, assessing the condition of tooth surfaces and the presence of caries according to the International Caries Detection and Assessment System (ICDAS II) in a very similar way to previous researchers [26].

After the patient selection, permanent first molars with deep pits and fissures, which were at risk of caries, were selected. To assess caries risk in children, the authors used the Caries Management by Risk Assessment (CAMBRA) system [27]. All permanent first molars underwent professional brushing using prophylactic brushes and fluoride-free toothpaste. The examination was performed visually and tactilely on clean and moist/dry teeth (following the recommendations of the ICDAS II classification). A probe with a ball-end was used to remove any remaining bacterial plaque and to check the contour of the occlusal surface, the presence or absence of carious lesions, cavities, and the existence of sealants.

Each occlusal surface was examined and received classification ranging from 0 up to 6 according to ICDAS II criteria [26] (Table 1):

Following this classification, only molars falling into the category of caries-free teeth and those with non-cavitated dentin carious lesions were selected and included in the study as follows:

- Molars without carious lesions, categorized according to the ICDAS II classification as code 0, constituted the Control group.
- Molars with extensive non-cavitated carious lesions reaching the dentin level, categorized according to the ICDAS II classification as code 3, constituted the Study group.

Molars with hypoplasia, fluorosis, other developmental anomalies, those with approximal carious lesions, sealants, restorations, or those experiencing sensitivity to any type of stimulus were excluded from the study. Additionally, molars with carious lesions limited to the enamel (ICDAS II, code 1 and 2) and molars with carious lesions in the dentin that had already resulted in cavities (ICDAS II, code 4, 5, and 6) were also excluded. All molars included in the Study group underwent radiological examination for a double confirmation of the extent of dentin carious lesions. This examination was conducted within the dental imaging department of the Dental Medicine Simulation Centre at our Faculty.

All participating patients in the study were instructed to maintain proper oral and dietary hygiene. The recommended manual brushing technique was the Bass technique, which has been widely accepted in the literature for its enhanced effectiveness in plaque removal. For those who preferred to use an electric toothbrush, correct usage was demonstrated. Instruction was conducted using models available in the Preventive and Community Dentistry Department. Regarding dietary hygiene, children were informed about the principles of a proper diet, focusing on limiting sugar intake and replacing it with healthy snacks whenever possible.

The molars included in the two groups were sealed with a light-curing white composite-resin fissure sealant with fluoride-releasing properties, Helioseal F™, Ivoclar Vivadent Schaan, Liechtenstein. All procedures were carried out with the assistance of a trained chair-side clinical assistant using the “four-handed technique”. The application steps for the material were identical for both groups. According to the manufacturer’s instructions, these steps were as follows: professional tooth brushing, rinsing with water, drying with air/water spray, isolation with cotton rolls and further drying with air, application of the demineralizing agent—37% phosphoric acid for 30 s, rinsing with water and drying with air, checking the etched tooth surface (opaque, matte appearance), applying the bonding agent, light-curing the bonding, applying the sealant, light-curing it for 20 s, and checking marginal adaptation and occlusion.

**Figure 1:**
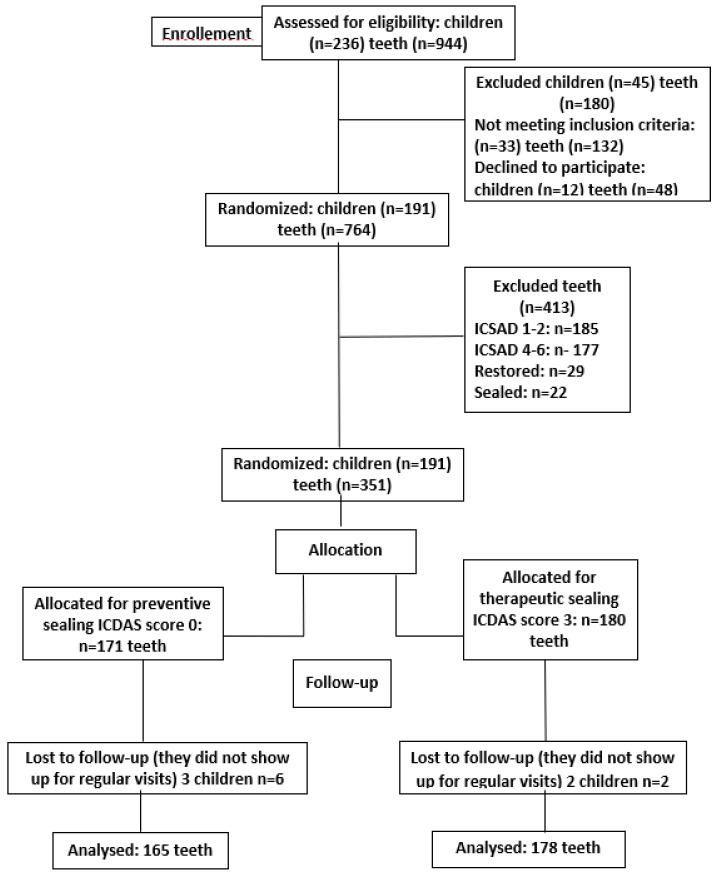
Consort flow diagram used the study.

Periodic check-ups were conducted at 6- and 12-month intervals. During each follow-up visit, for each molar included in the study, the clinical retention of the material (Control and Study groups) and the incidence of new carious lesions for the Control group, or the evolution of existing lesions for the Study group, were assessed (Figure 1). To evaluate the sealant material’s retention, classic criteria used in the specialty literature—Simonsen’s criteria—were employed as follows:

I—sealant retained entirely;
II—sealant partially retained;
III—sealant lost completely [28].

In cases where the sealant was completely retained, no intervention was required. In situations where it was partially lost, after excluding a cavitated carious process, the sealant was reapplied to the appropriate areas. When it was lost entirely, a new seal was applied, again after excluding a cavitated carious process. In the case where a carious lesion has progressed and resulted in a cavity, the recommendation was to use the conventional therapeutic technique. The integrity and marginal adaptation of the sealant were assessed through visual and tactile examination.

At the 12-month interval, molars from the experimental Study group, underwent a follow-up radiological examination to evaluate the progression of the carious lesion from this perspective as well. These radiographs were compared with the initial radiographs. Carious lesion progression was recorded when the final radiograph showed an increase in the lesion (radiolucency) compared to the initial radiograph, either in the occlusal-apical or mesial-distal direction. The cessation of the carious process was noted when there was no change in both dimensional planes.

### 2.1. Sample Size Determination

The required sample size was determined to be 703 teeth using G-power software™ for Windows, Heinrich Heine University, Dusseldorf, Germany, with a power of 95% (α = 0.05, β = 0.05).

### 2.2. Statistical Analysis

The results obtained from the examinations were recorded using GraphPad Prism™ V6.01 software for Windows™ 2017. For the evaluation of categorical data, the authors employed Fisher’s exact test and the chi-squared test. The selected significance level was set at 0.05, and *p* was considered statistically significant when *p* ≤ 0.05.

## 3. Results

Out of the 236 examined children, 45 were excluded either because they did not fall into the high-caries risk group according to the CAMBRA classification or because their four permanent first molars had already been sealed or restored, were affected by hypoplasia, fluorosis, or other developmental anomalies, or displayed carious lesions that, according to the ICDAS II classification, scored at least 4. Therefore, the authors included 191 children and 764 permanent first molars in the study. These were categorized according to the ICDAS classification as follows: ICDAS score 0–171 (22.38%) molars, ICDAS score 1–2–185 (24.21%) molars, ICDAS score 3–180 (23.56%) molars, ICDAS score 4–6–177 (23.16%) molars. Twenty-nine molars (3.79%) had fillings, and 22 (2.87%) were sealed. So, the initial sample included 171 caries-free molars, forming the Control group, and 180 molars categorized according to the ICDAS classification with a score of 3, forming the Study group.

At the 12-month follow-up, four children did not show up, and we lost eight molars, six of which were classified as ICDAS score 0 and 2 as ICDAS score 3. Thus, the final sample of the study included 165 molars classified as ICDAS score 0 and 178 molars classified as ICDAS score 3, which were sealed and assessed.

The follow-up intervals were 6 and 12 months. All the obtained data were systematized in tables.

Both the 6-month (*p* = 0.0050) and 12-month (*p* = 0.0001) follow-up intervals showed statistically significant differences in terms of sealant retention between the two types of sealed teeth: sound (ICDAS 0) and decayed (ICDAS 3) (Table 2 and Table 3).

Additionally, the 6-month (*p* = 0.0306) and 12-month (*p* = 0.0029) follow-up assessments revealed statistically significant differences between the two types of sealed teeth regarding the development of carious lesions (Table 4 and Table 5).

Seven of the molars initially classified with code 0 according to ICDAS II classification required the reapplication of a new sealant at the 6-month follow-up assessments. Two of these lesions progressed to code 1, while the other five lesions did not progress at all. Nineteen of the molars initially classified with code 0 according to ICDAS II classification required the reapplication of a new sealant at the 12-month follow-up assessments. Five of these lesions progressed to code 1, and three progressed to code 2. The other 11 lesions did not progress at all. None of these lesions progressed to code 3, 4, 5, or 6.

Twenty-two of the carious lesions initially classified as code 3 according to ICDAS II criteria required the reapplication of sealants at the 6-month assessments. Three carious lesions required the application of fillings due to their transformation into lesions classified as code 4. The remaining lesions that retained their sealant intact did not require further intervention. Twenty-nine of the carious lesions initially classified as code 3 according to ICDAS II criteria required the reapplication of sealants at the 12-month assessments. Twenty-seven carious lesions required the application of fillings due to their transformation into lesions classified as code 4. Similarly, the rest of the sealed lesions, which maintained their sealant intact, did not require further intervention. None of these lesions progressed to code 4, 5, or 6.

At the 12-month interval, radiological examination of molars from the Study group, where the sealants were retained, revealed the cessation of the carious process in both dimensional planes.

## 4. Discussion

The study explores an important clinical intervention in the field of dentistry, particularly focusing on the treatment of early-stage tooth decay. The study evaluates resin-based composite sealants, a material commonly used in dental restorations, in treating non-cavitated dentin carious lesions, which are classified as ICDAS 3. These are early carious lesions that have not yet formed a cavity but show visible signs of enamel breakdown with localized enamel breakdown and underlying shadowing from dentin [29]. These early interventions are crucial for preventing the progression of tooth decay, potentially reducing the need for more invasive treatments later. Resin-based composite sealants are noteworthy because, unlike traditional sealants that mainly act as a physical barrier, resin-based composites might provide additional benefits such as better adherence and aesthetic qualities [30]. They might also offer active cariostatic properties due to the release of fluoride or other agents, enhancing their effectiveness in arresting caries development. The most crucial criterion for evaluating the success of a composite resin-based sealant is its retention in the grooves and fissures where it was applied [31]. Studies analyzing this aspect have concluded that the retention rate decreases over time. For example, a recent meta-analysis study found the retention rate of light-polymerizing sealants to be 83.1%, 68.4%, and 57.8% at year 2, 3, and 5 of the check-ups periods [32]. Since the partial or total loss of the sealant leads to a loss of its ability to protect against carious lesions, it needs to be replaced [33]. These findings underscore the need for regular check-ups and reintervention [34]. In the case of using sealants for therapeutic purposes, ensuring adequate retention is an even greater challenge. Even when the proper isolation of the tooth from saliva is achieved [35,36], demineralized enamel and underlying dentin can compromise polymerization shrinkage, marginal leakage, and the adhesion of the sealing material [37,38].

In the present study, the retention rate for the application of the sealant for therapeutic purposes (Study group) is much lower and statistically significantly different (*p* = 0.0001) than that for the application of the sealant for preventive purposes (Control group). Comparing with the specialized literature, the authors found that the retention of the sealant in our study 1-year post-application is similar to the retention of sealants applied for preventive purposes 3 years post-application [32]. This massive loss of the sealant applied for therapeutic purposes raises questions about its long-term effectiveness. The premature loss of sealants in such a short time interval has led not only to many resealing but also to the progression of sealed carious lesions that lost the sealant, transforming them into a percentage of 48% cavitated carious lesions that have a classic indication for treatment. This premature loss of the sealant applied to these types of carious lesions has been reported by other authors as well [35,39] and represents one of the strengths of the present research.

In the case of teeth sealed for therapeutic purposes that did not lose the sealant during the studied time interval, we did not record the progression of the underlying carious lesion, neither in clinical nor radiological evaluation. This decision is supported by numerous studies in the specialized literature in recent decades. Their results support that as long as the sealant remains intact and provides a hermetic closure, carious lesions are isolated from the external environment, and access to nutrients is halted. This leads to a dramatic reduction in the number of viable bacteria and the survival of less virulent bacterial species [40,41,42], slowing down or stopping the progression of the underlying carious lesion [43,44]. However, in the literature, there are also studies that contradict this hypothesis of stopping the progression of grafted carious lesions already at the dentin level. According to this research, sealants cannot penetrate the depth of carious lesions, leaving empty spaces where microorganisms can develop and continue their demineralizing activity [45]. Unlike saccharolytic bacteria associated with enamel caries, the bacteria present in dentin carious lesions are proteolytic bacteria with the ability to degrade the protein component of dentin to provide themselves with nutrients, without the need for external nutritional input [46,47]. In accordance with these studies, the lack of progression of dentin carious lesions in our study that retained the sealant may be due to the short time interval in which they were followed. Moreover, the significant loss of sealants in this period could be explained by the progression of carious lesions followed by the collapse of the undermined enamel [44], transforming them into cavitated lesions, rather than the progression of the carious lesion due to sealant loss.

Knowing that the retention rate decreases over time, the early and large loss of sealants, as well as the transformation of non-cavitated dentin lesions into cavitated lesions within a short period of time—12 months—raises serious questions about the long-term effectiveness of dental sealant applied to this type of carious lesion. On the one hand, the difficulty of accessing these carious lesions with the impossibility of completely removing the bacterial plaque and the faithful adaptation of the sealant facilitate the continuation of demineralizing activity and their progression [37,38,45]. On the other hand, the enamel and dentin to which the sealant is applied are already partially demineralized, compromising polymerization shrinkage, marginal leakage, adhesion, and retention [37,38].

Although there is currently no well-documented scientific evidence that sealant materials containing fluoride are more effective in preventing and arresting caries [48,49,50], the authors considered it useful to use such a sealant because fluoride is an element whose ability to reduce bacterial metabolic activity, inhibit demineralization processes, and facilitate mineralization processes is supported by numerous specialized studies [51,52]. Recent studies [52,53] suggest that various operative factors, including moisture control, surface preparation, and the application of dental adhesive, affect the clinical outcomes, and there are data [54] indicating better results in terms of the retention rate of the resin-based fissure sealant, but this cannot be the basis for concluding that this type of material is superior for the stated task. Further research is undoubtedly needed to determine which material is best suited for application as a fissure sealant [55].

### Limitations of the Study

It is mandatory to mention the limitations of the study, which include the small study group, the specific population (school children in Târgu Mureș), and the short observation period. This study is ongoing, and further results will be published in the future.

## 5. Conclusions

Composite resin-based sealants demonstrate effectiveness in both arresting early-stage carious lesions (ICDAS 3) and preventing the onset of caries in healthy teeth (ICDAS 0). This dual efficacy highlights the versatility of the sealant as a valuable tool in dental care, supporting its use not only in patients with initial signs of decay but also as a preventive measure in caries-free individuals.

The durability and integrity of the sealant over the 12-month period are critical for its effectiveness. Proper adherence and lack of degradation are essential for sustained protective action against caries. This underscores the importance of material choice and application technique in clinical practice, ensuring that sealants remain an effective part of caries management strategies.

## Figures and Tables

**Table 1:** International Caries Detection and Assessment System.

code 0	sound tooth surface; no evidence of caries after prolonged air drying (5 s)
code 1	first visual change in enamel: opacity or discoloration (white or brown) is visible at the entrance to the pit or fissure after prolonged air drying, which is not or hardly seen on wet surface
code 2	distinct visual change in enamel: opacity or discoloration distinctly visible at the entrance to the pit and fissure when wet, lesion must still be visible when dry
code 3	localized enamel breakdown due to caries with no visible dentine or underlying shadow: opacity or discoloration wider than the natural fissure/fossa when wet and after prolonged air drying
code 4	underlying dark shadow from dentine with or without localized enamel breakdown
code 5	distinct cavity with visible dentine: visual evidence of demineralization and dentine exposed

**Table 2:** Retention based on Simonsen’s criteria at the 6-month follow-up.

6 Months	I	II	III	Total	*p* Value
code 0 ICDAS	95.75% (158)	0	4.24% (7)	165	
code 3 ICDAS	85.95% (153)	2.24% (4)	11.79% (21)	178	*p* = 0.0050

**Table 3:** Retention based on Simonsen’s criteria at the 12-month follow-up.

12 Months	I	II	III	Total	*p* Value
code 0 ICDAS	88.48% (146)	1.81% (3)	9.69% (16)	165	
code 3 ICDAS	64.41% (122)	7.86% (14)	23.59% (42)	178	*p* = 0.0001

**Table 4:** Presence of carious lesions at 6 months.

6 Months	Yes	No	Total	*p* Value
code 0 ICDAS	0	165	165	
code 3 ICDAS	3.37% (6)	96.62% (172)	178	*p* = 0.0306

**Table 5:** Presence of carious lesions at 12 months.

12 Months	Yes	No	Total	*p* Value
code 0 ICDAS	4.84% (8)	95.15% (157)	165	
code 3 ICDAS	15.16% (27)	84.83% (151)	178	*p* =0.0029

## Data Availability

All data regarding this manuscript can be checked with corresponding authors.

## References

[1] Ramos-Gomez F., Kinsler J., Askaryar H. (2020). Understanding oral health disparities in children as a global public health issue: How dental health professionals can make a difference. J. Public Health Policy.

[2] Peres M.A., Macpherson L.M.D., Weyant R.J., Daly B., Venturelli R., Mathur M.R., Listl S., Celeste R.K., Guarnizo-Herreño C.C., Kearns C. (2019). Oral diseases: A global public health challenge. Lancet.

[3] Qin X., Zi H., Zeng X. (2022). Changes in the global burden of untreated dental caries from 1990 to 2019: A systematic analysis for the Global Burden of Disease study. Heliyon.

[4] Splieth C. (2021). Innovation in Preventive Dentistry.

[5] Warreth A. (2023). Dental Caries and Its Management. Int. J. Dent..

[6] Schwendicke F., Frencken J.E., Bjørndal L., Maltz M., Manton D.J., Ricketts D., Van Landuyt K., Banerjee A., Campus G., Doméjean S. (2016). Managing Carious Lesions: Consensus Recommendations on Carious Tissue Removal. Adv. Dent. Res..

[7] Keyes P.H. (1960). The infectious and transmissible nature of experimental dental caries. Findings and implications. Arch. Oral Biol..

[8] Innes N.P.T., Chu C.H., Fontana M., Lo E.C.M., Thomson W.M., Uribe S., Heiland M., Jepsen S., Schwendicke F. (2019). A Century of Change towards Prevention and Minimal Intervention in Cariology. J. Dent. Res..

[9] Philip N., Suneja B. (2023). The revolutionary evolution in carious lesion management. J. Conserv. Dent..

[10] Philip N. (2019). State of the Art Enamel Remineralization Systems: The Next Frontier in Caries Management. Caries Res..

[11] Kidd E., Fejerskov O. (2013). Changing concepts in cariology: Forty years on. Dent. Update.

[12] Schwendicke F. (2017). Contemporary concepts in carious tissue removal: A review. J. Esthet. Restor. Dent..

[13] Mertz-Fairhurst E.J., Schuster G.S., Fairhurst C.W. (1986). Arresting caries by sealants: Results of a clinical study. J. Am. Dent. Assoc..

[14] Borges B.C., De Souza R.F., Dantas R.F., De Araújo A., De Assunção P. (2012). Efficacy of a non-drilling approach to manage non-cavitated dentin occlusal caries in primary molars: A 12-month randomized controlled clinical trial. Int. J. Paediatr. Dent..

[15] Innes N.P., Ricketts D., Chong L.Y., Keightley A.J., Lamont T., Santamaria R.M. (2015). Preformed crowns for decayed primary molar teeth. Cochrane Database Syst. Rev..

[16] Nyvad B., Machiulskiene V., Baelum V. (1999). Reliability of a new caries diagnostic system differentiating between active and inactive caries lesions. Caries Res..

[17] Kidd E. (2011). The implications of the new paradigm of dental caries. J. Dent..

[18] Pitts N.B., Zero D.T., Marsh P.D., Ekstrand K., Weintraub J.A., Ramos-Gomez F., Tagami J., Twetman S., Tsakos G., Ismail A. (2017). Dental caries. Nat. Rev. Dis. Primers.

[19] Cvikl B., Moritz A., Bekes K. (2018). Pit and Fissure Sealants-A Comprehensive Review. Dent. J..

[20] Cabalén M.B., Molina G.F., Bono A., Burrow M.F. (2022). Nonrestorative Caries Treatment: A Systematic Review Update. Int. Dent. J..

[21] Bereșescu L., Păcurar M., Bica C.I., Vlasa A., Stoica O.E., Dako T., Petcu B., Esian D. (2022). The Assessment of Sealants’ Effectiveness in Arresting Non-Cavitated Caries Lesion—A 24-Month Follow-Up. Healthcare.

[22] Slayton R.L., Urquhart O., Araujo M.W.B., Fontana M., Guzmán-Armstrong S., Nascimento M.M., Nový B.B., Tinanoff N., Weyant R.J., Wolff M.S. (2018). Evidence-based clinical practice guideline on nonrestorative treatments for carious lesions: A report from the American Dental Association. J. Am. Dent. Assoc..

[23] Berdouses E.D., Michalaki M., Tsinidou K., Vlachou A., Pantazis N., Oulis C.J. (2021). Effectiveness of fissure sealants on initial caries lesions (ICDAS 1-3) of permanent molars: A 4-year follow-up. Eur. J. Paediatr. Dent..

[24] Lam P.P., Sardana D., Lo E.C., Yiu C.K. (2021). Fissure sealant in a nutshell. Evidence-based meta-evaluation of sealant’ effectiveness in caries prevention and arrest. J. Evid. Based Dent. Pract..

[25] Urquhart O., Tampi M.P., Pilcher L., Slayton R.L., Araujo M.W.B., Fontana M., Guzmán-Armstrong S., Nascimento M.M., Nový B.B., Tinanoff N. (2019). Nonrestorative Treatments for Caries: Systematic Review and Network Meta-analysis. J. Dent. Res..

[26] International Caries Detection and Assessment System (ICDAS) Coordinating Committee. https://www.iccmsweb.com/uploads/asset/592848be55d87564970232.pdf.

[27] Caries Risk Assessment-California Dental Association. https://www.cda.org/wp-content/uploads/2023/01/journal_102007.pdf.

[28] Simonsen R.J. (1991). Retention and Effectiveness of Dental Sealant After 15 Years. J. Am. Dent. Assoc..

[29] Ahovuo-Saloranta A., Forss H., Walsh T., Nordblad A., Mäkelä M., Worthington H.V. (2017). Pit and fissure sealants for preventing dental decay in permanent teeth. Cochrane Database Syst. Rev..

[30] Beresescu L., Pacurar M., Vlasa A., Stoica A.M., Dako T., Petcu B., Eșian D. (2022). Comparative Assessment of Retention and Caries Protective Effectiveness of a Hydrophilic and a Conventional Sealant—A Clinical Trial. Children.

[31] Wright J.T., Tampi M.P., Graham L., Estrich C., Crall J.J., Fontana M., Gillette E.J., Nový B.B., Dhar V., Donly K. (2016). Sealants for preventing and arresting pit-and-fissure occlusal caries in primary and permanent molars: A systematic review of randomized controlled trials-a report of the American Dental Association and the American Academy of Pediatric Dentistry. J. Am. Dent. Assoc..

[32] Kühnisch J., Bedir A., Lo Y.F., Kessler A., Lang T., Mansmann U., Heinrich-Weltzien R., Hickel R. (2020). Meta-analysis of the longevity of commonly used pit and fissure sealant materials. Dent. Mater..

[33] Jodkowska E. (2008). Efficacy of pit and fissure sealing: Long-term clinical observations. Quintessence Int..

[34] Desai H., Stewart C.A., Finer Y. (2021). Minimally Invasive Therapies for the Management of Dental Caries—A Literature Review. Dent. J..

[35] Qvist V., Borum M.K., Møller K.D., Andersen T.R., Blanche P., Bakhshandeh A. (2017). Sealing Occlusal Dentin Caries in Permanent Molars: 7-Year Results of a Randomized Controlled Trial. JDR Clin. Trans. Res..

[36] Borges B.C., de Souza Borges J., Braz R., Montes M.A., de Assunção Pinheiro I.V. (2012). Arrest of non-cavitated dentinal occlusal caries by sealing pits and fissures: A 36-month, randomised controlled clinical trial. Int. Dent. J..

[37] Hevinga M., Opdam N., Frencken J., Bronkhorst E., Truin G. (2007). Microleakage and sealant penetration in contaminated carious fissures. J. Dent..

[38] Hevinga M., Opdam N., Frencken J., Bronkhorst E., Truin G. (2008). Can Caries Fissures be Sealed as Adequately as Sound Fissures?. J. Dent. Res..

[39] Machiulskiene V., Campus G., Carvalho J.C., Dige I., Ekstrand K.R., Jablonski-Momeni A., Maltz M., Manton D.J., Martignon S., Martinez-Mier E.A. (2020). Terminology of Dental Caries and Dental Caries Management: Consensus Report of a Workshop Organized by ORCA and Cariology Research Group of IADR. Caries Res..

[40] Orhan A.I., Oz F.T., Ozcelik B., Orhan K. (2008). A clinical and microbiological comparative study of deep carious lesion treatment in deciduous and young permanent molars. Clin. Oral Investig..

[41] Paddick J.S., Brailsford S.R., Kidd E.A., Beighton D. (2005). Phenotypic and genotypic selection of microbiota surviving under dental restorations. Appl. Environ. Microbiol..

[42] Going R.E., Loesche W.J., Grainger D.A., Syed S.A. (1978). The viability of microorganisms in carious lesions five years after covering with a fissure sealant. J. Am. Dent. Assoc..

[43] Schmid-Schwap M., Graf A., Preinerstorfer A., Watts D.C., Piehslinger E., Schedle A. (2011). Microleakage after thermocycling of cemented crowns—A meta-analysis. Dent. Mater..

[44] Fontana M., Innes N. (2018). Sealing Carious Tissue Using Resin and Glass-Ionomer Cements. Monogr. Oral Sci..

[45] Kielbassa A.M., Ulrich I., Schmidl R., Schüller C., Frank W., Werth V.D. (2017). Resin infiltration of deproteinised natural occlusal subsurface lesions improves initial quality of fissure sealing. Int. J. Oral Sci..

[46] Pugach M.K., Strother J., Darling C.L., Fried D., Gansky S.A., Marshall S.J., Marshall G.W. (2009). Dentin caries zones: Mineral, structure, and properties. J. Dent. Res..

[47] Fusayama T., Kurosaki N. (1972). Structure and removal of carious dentin. Int. Dent. J..

[48] Yildiz E., Dörter C., Efes B., Koray F. (2004). A comparative study of two fissure sealants: A 2-year clinical follow-up. J. Oral Rehabil..

[49] Muller-Bolla M., Courson F., Lupi-Pégurier L., Tardieu C., Mohit S., Staccini P., Velly A.M. (2018). Effectiveness of Resin-Based Sealants with and without Fluoride Placed in a High Caries Risk Population: Multicentric 2-Year Randomized Clinical Trial. Caries Res..

[50] Cury J.A., de Oliveira B.H., dos Santos A.P., Tenuta L.M. (2016). Are fluoride releasing dental materials clinically effective on caries control?. Dent. Mater..

[51] Li F., Li F., Wu D., Ma S., Gao J., Li Y., Xiao Y., Chen J. (2011). The Effect of an Antibacterial Monomer on the Antibacterial Activity and Mechanical Properties of a Pit-and-Fissure Sealant. J. Am. Dent. Assoc..

[52] Wiegand A., Buchalla W., Attin T. (2007). Review on fluoride-releasing restorative materials—Fluoride release and uptake characteristics, antibacterial activity and influence on caries formation. Dent. Mater..

[53] Ng T.C., Chu C.H., Yu O.Y. (2023). A concise review of dental sealants in caries management. Front. Oral Health.

[54] Wnuk K., Świtalski J., Miazga W., Tatara T., Religioni U., Gujski M. (2023). Evaluation of the effectiveness of prophylactic sealing of pits and fissures of permanent teeth with fissure sealants—Umbrella review. BMC Oral Health..

[55] Piszko A., Piszko P.J., Lubojański A., Grzebieluch W., Szymonowicz M., Dobrzyński M. (2023). Brief Narrative Review on Commercial Dental Sealants—Comparison with Respect to Their Composition and Potential Modifications. Materials.

